# Supplementary material for: Comparative transcriptomics reveals highly conserved regional programs between porcine and human colonic enteric nervous system
Source: Commun Biol. 2023 Jan 24;6:98. doi: 10.1038/s42003-023-04478-x (PMC9872754; doi:10.1038/s42003-023-04478-x)
Supplement: Supplementary file 2 — Supplementary Information [file 42003_2023_4478_MOESM2_ESM.pdf]

Supplementary information (Figures and Tables)

Supplementary Figure 1:

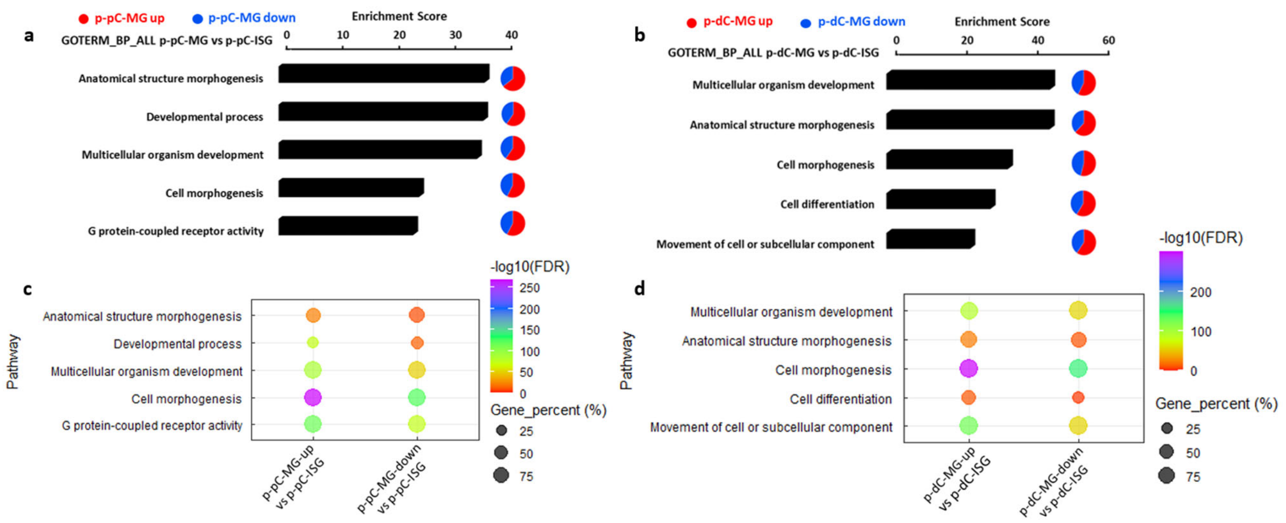

Supplementary Fig. 1 Comparison of pathway enrichment between myenteric ganglia (MG) and inner submucosal ganglia (ISG) in porcine proximal or distal colon (p-pC, p-dC). a, b Enrichment of top five biological processes (BPs). The pie charts show the percentage of visible genes involved in the specified BPs. Red and blue color represents the genes with upregulated and downregulated expression levels in MG in comparison with ISG, respectively. X-axis marks enrichment score, with the significance cut-off marked by the vertical white line (*p*-value < 0.05). c, d The bubble plots show the gene percentage and enrichment of the top BPs. Circle size represents the ratio of the number of pathway-specific differentially expressed genes (DEG) and the number of total DEG in each DEG list (Gene_percent). Color represents a -log10(FDR) distribution from big (orange) to small (purple). Up, upregulated expression. Down, downregulated expression.

Supplementary Figure 2:

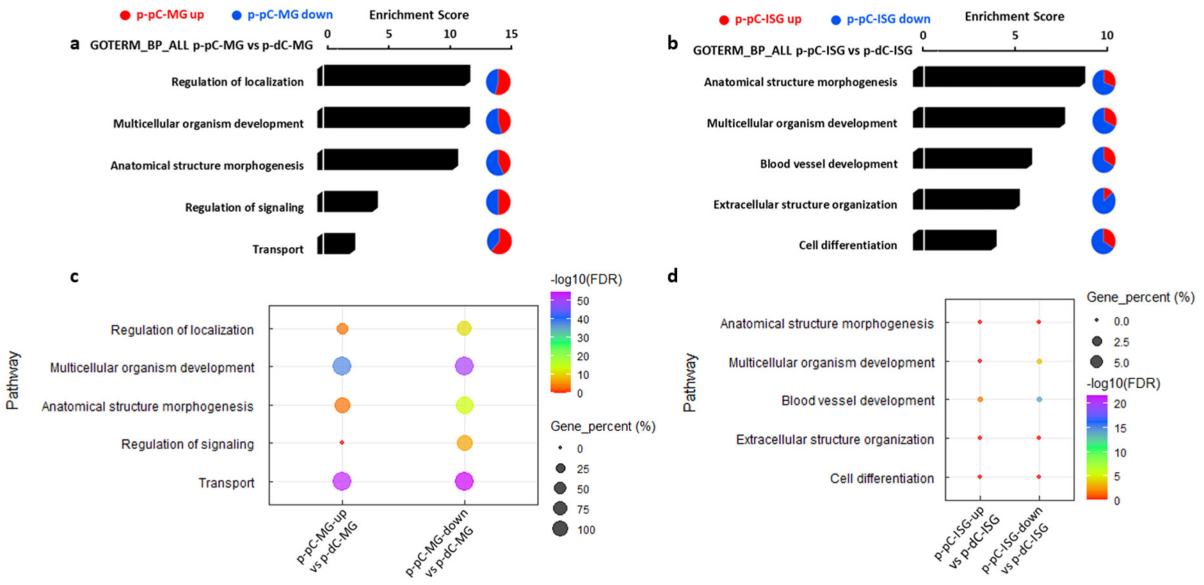

Supplementary Fig. 2 Comparison of pathway enrichment in myenteric ganglia (MG) or inner submucosal ganglia (ISG) between porcine proximal and distal colon (p-pC, p-dC). a, b Enrichment of top five biological processes (BPs). The pie charts show the percentage of visible genes involved in the specified BPs. Red and blue color represents the genes with upregulated and downregulated expression levels in p-pC-MG or p-pC-ISG in comparison with p-dC-MG or p-dC-ISG, respectively. X-axis marks enrichment score, with the significance cut-off marked by the vertical white line (p-value < 0.05). c, d The bubble plots show the gene percentage and enrichment of the top BPs. Circle size represents the ratio of the number of pathway-specific differentially expressed genes (DEG) and the number of total DEG in each DEG list (Gene_percent). Color represents a -log10(FDR) distribution from big (orange) to small (purple). Up, upregulated expression. Down, downregulated expression.

Supplementary Figure 3:

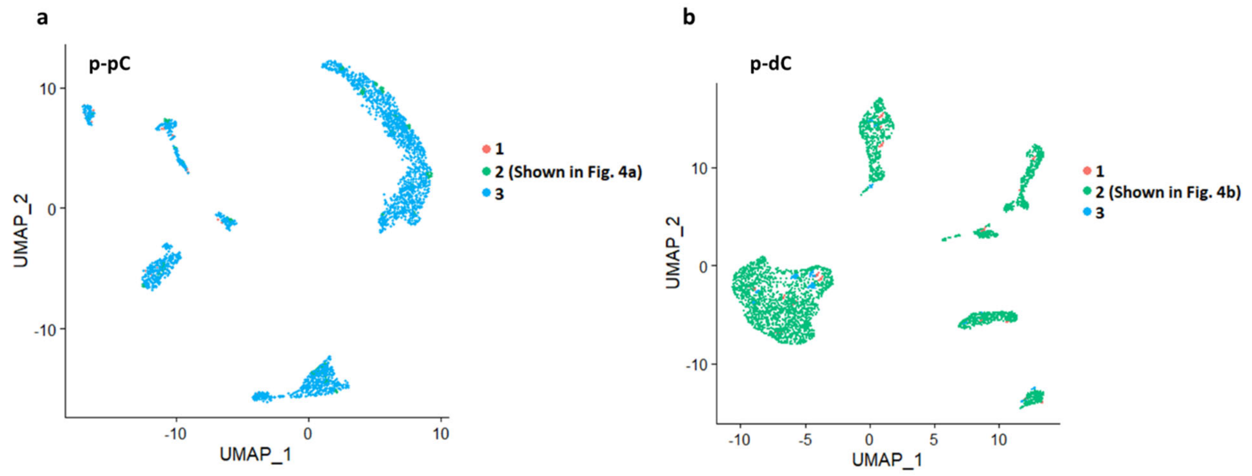

Supplementary Fig. 3 Joint visualization of single-cell RNA sequencing data from three individuals of porcine proximal colon (p-pC) (a) and distal colon (p-dC) (b), related to Figure 4.

Supplementary Figure 4:

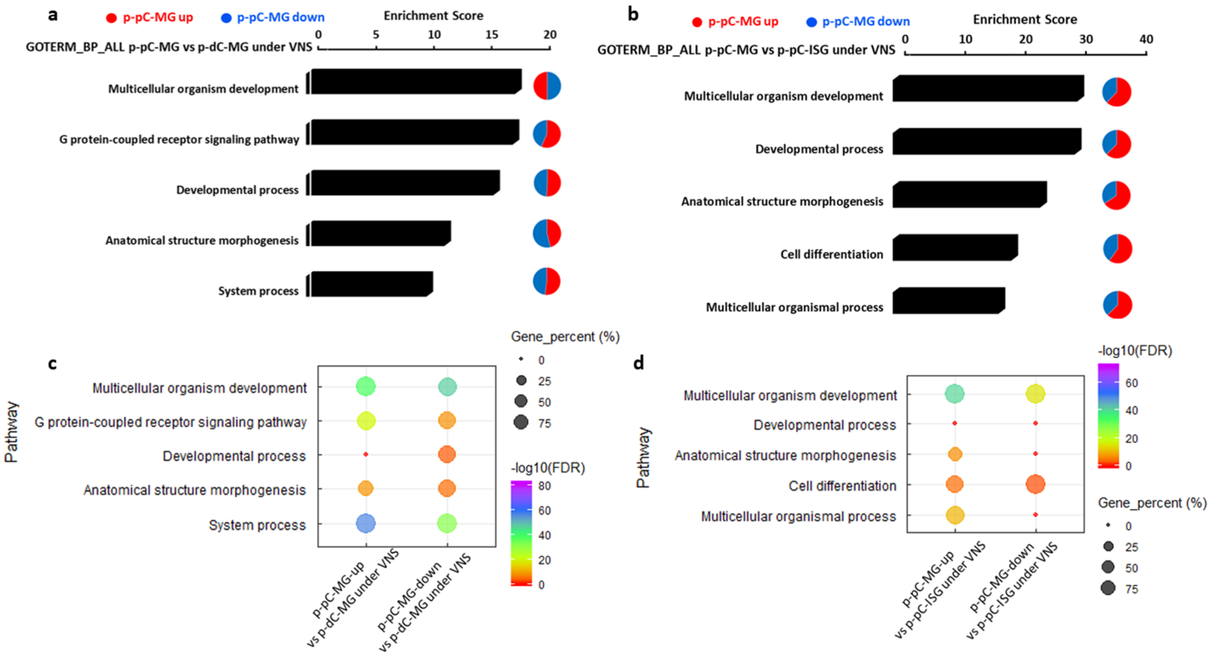

Supplementary Fig. 4 Comparison of pathway enrichment in myenteric ganglia (MG) between porcine proximal and distal colon (p-pC, p-dC) and between p-pC-MG and inner submucosal ganglia (ISG) in p-pC with vagal nerve stimulation (VNS). a, b Enrichment of top five biological processes (BPs). The pie charts show the percentage of visible genes involved in the specified BPs. Red and blue color represents the genes with upregulated and downregulated expression levels in p-pC-MG in comparison with p-dC-MG or p-pC-ISG, respectively. X-axis marks enrichment score, with the significance cut-off marked by the vertical white line (*p*-value < 0.05). c, d The bubble plots show the gene percentage and enrichment of the top BPs. Circle size represents the ratio of the number of pathway-specific differentially expressed genes (DEG) and the number of total DEG in each DEG list (Gene_percent). Color represents a -log10(FDR) distribution from big (orange) to small (purple). Up, upregulated expression. Down, downregulated expression.

Supplementary Figure 5:

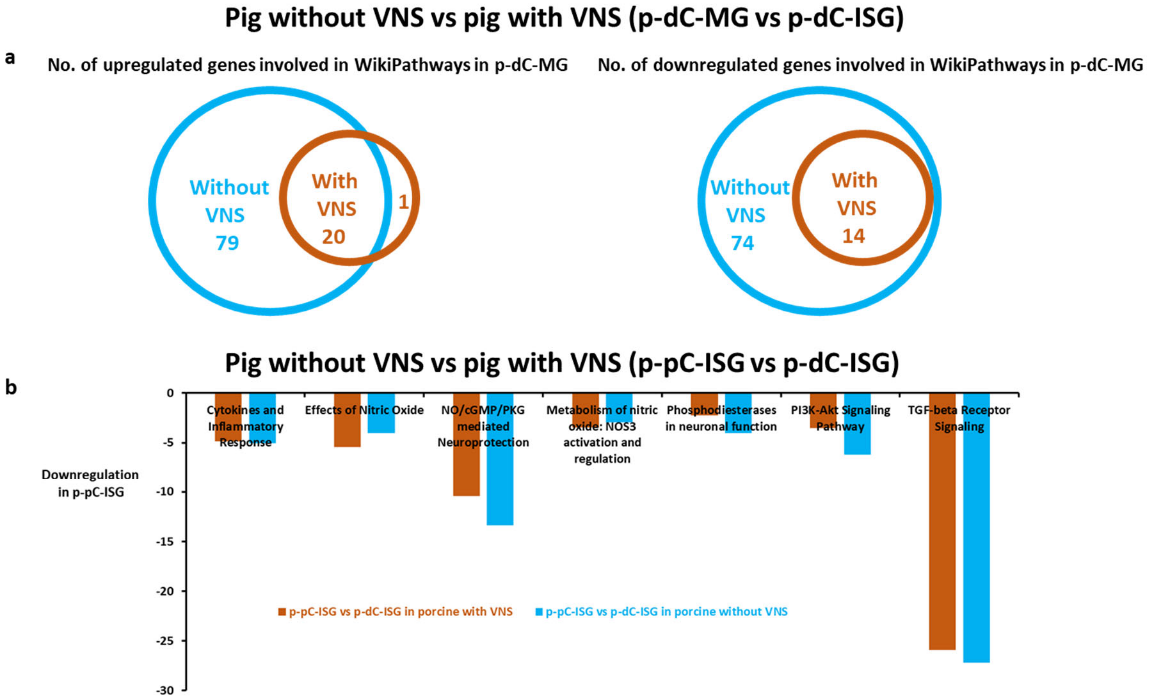

Supplementary Fig. 5 Comparison of pathway enrichment between myenteric ganglia (MG) and inner submucosal ganglia (ISG) in porcine distal colon (p-dC) (a) and between ISG in proximal colon (p-pC) and p-dC-ISG (b) with and without vagal nerve stimulation (VNS). The Venn diagram illustrates the numbers of the differentially expressed genes involved in WikiPathways (a) and the bar graph shows the difference in WikiPathways enrichment (b). The light blue color represents the difference without VNS and the orange color represents the difference with VNS. Fig. b shows the enrichment ratio of the same WikiPathway involving upregulated and downregulated genes in each list of differentially expressed genes based on the bulk RNA sequencing data. The light blue bars represent the enrichment ratio without VNS and the orange bars represent the enrichment ratio with VNS.

Supplementary Figure 6:

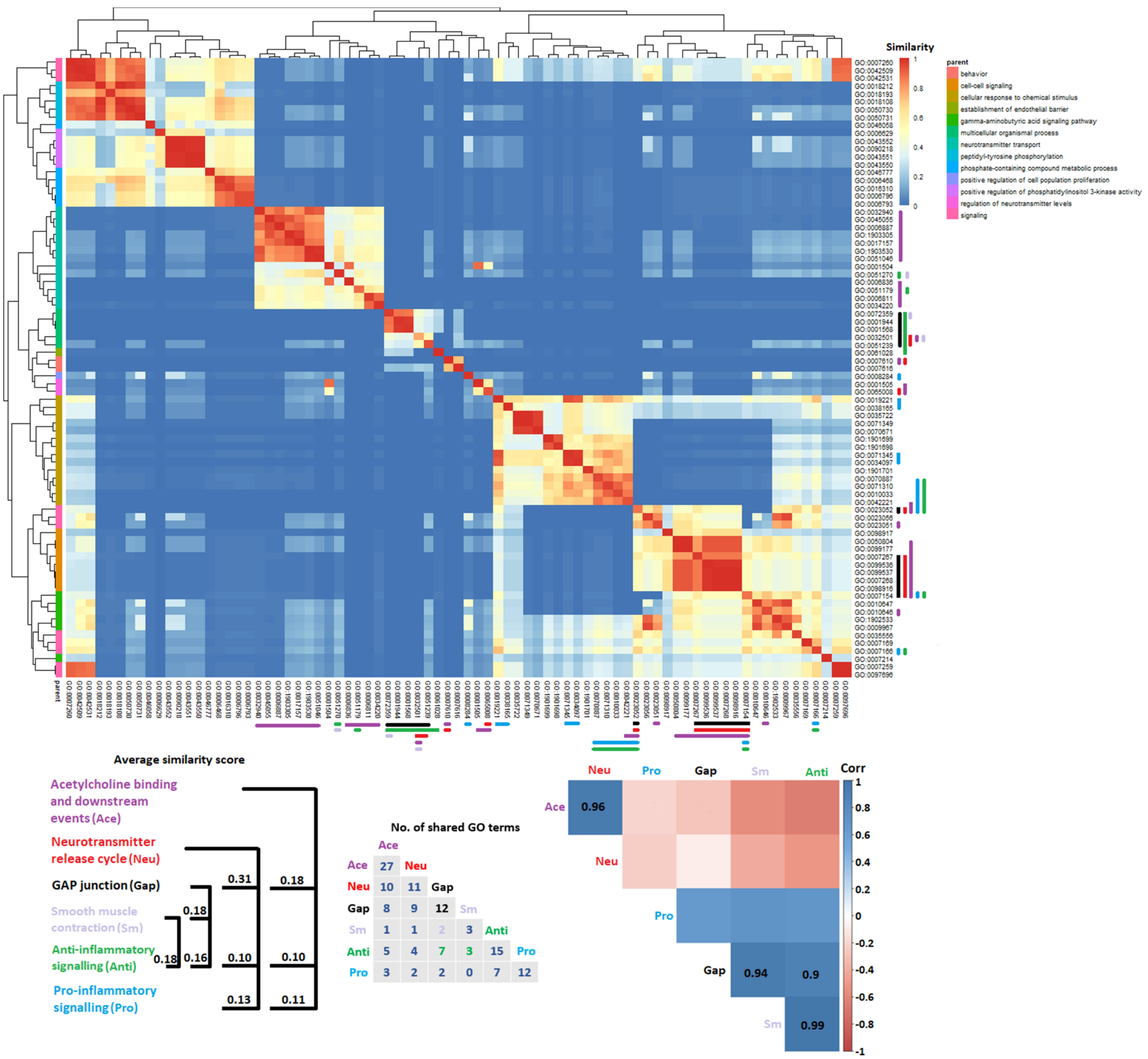

Supplementary Fig. 6 Heatmap represents the similarity matrix between Gene Ontology (GO) terms in comparison of myenteric ganglia (MG) and inner submucosal ganglia (ISG) in porcine proximal colon (p-pC) with vagal nerve stimulation (VNS). Only biological processes were selected for the analysis. Color represents a similarity distribution from big (red) to small (blue). The child GO terms were defined in a number of parent terms and WikiPathways of interest. The number of shared GO terms between WikiPathways of interest and average similarity score between the WikiPathways were also summarized. Pearson correlation analysis was performed based on pathway enrichment scores. Color represents a correlation distribution from positive (blue) to negative (red). The insignificant correlations were left blank. The color bars represent the specified child GO terms classified into designated clusters.

Supplementary Figure 7:

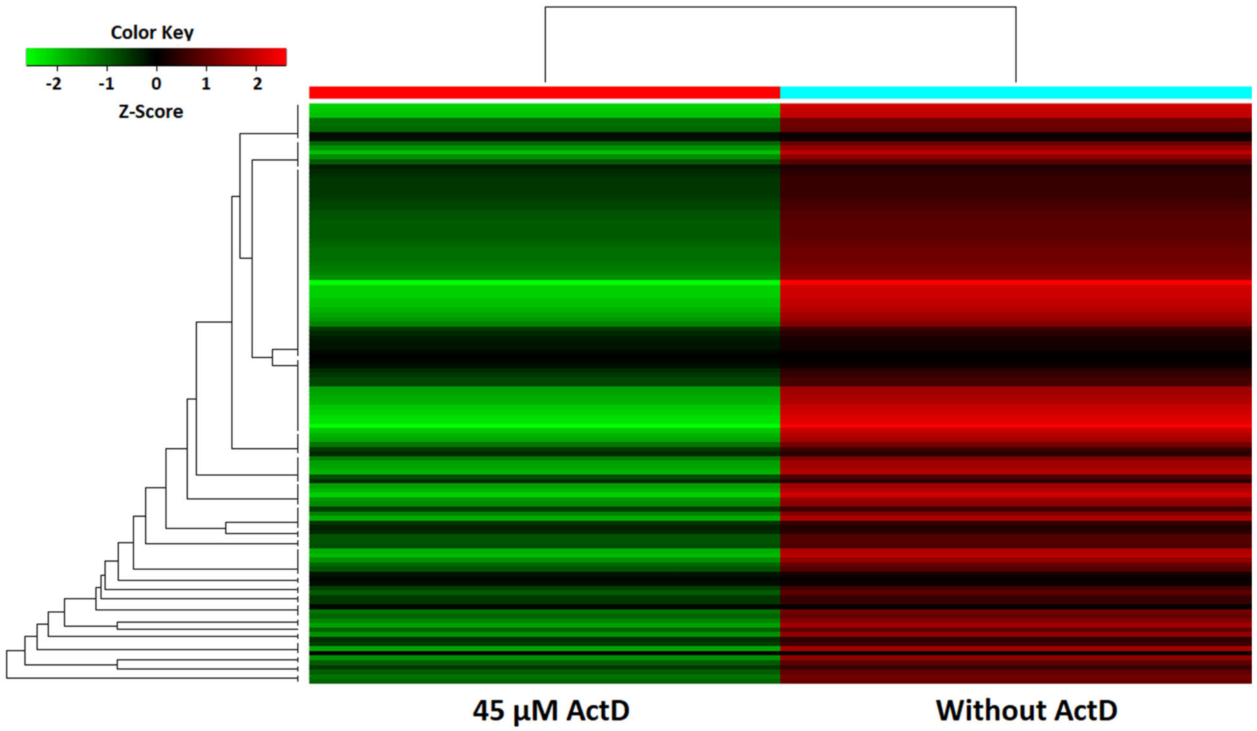

Supplementary Fig. 7 Heatmap showing that addition of Actinomycin D (ActD) suppresses artificially induced expression levels of immediate-early genes (IEGs) during single-cell dissociation. After cell suspension preparation from muscularis externa of porcine proximal colon, more than 1 million cells per sample were collected for bulk RNA-seq. The expression levels of total 127 IEGs were extracted. The gene expression was log_2 transformed and was displayed as colors ranging from red (high) to green (low) as shown in the key.

Supplementary Table 1:

Receptors and ligands expressed in neuronal and glial subsets

Receptor	Ligand
AGTR1/Cholinergic neurons	VEGFA/ Nitrergic neurons
AGTR1/Cholinergic neurons	ALB/SLC41A1_Glia
ITGB1/Cholinergic neurons	VEGFA/ Nitrergic neurons
ITGB1/Cholinergic neurons	ALB/SLC41A1_Glia
ITGB1/Cholinergic neurons	SPP1/SLC41A1_Glia
ITGB1/Cholinergic neurons	TGFB3/SLC41A1_Glia
LDLR/Cholinergic neurons	CLU/SPC24_Glia
LDLR/Cholinergic neurons	ALB/SLC41A1_Glia
VCL/Cholinergic neurons	ALB/SLC41A1_Glia
GPC4/Glutamatergic neurons	PCOLCE2/ Cholinergic neurons
LEPR/Glutamatergic neurons	IL7/SLC41A1_Glia
LEPR/Glutamatergic neurons	IGF1/SLC41A1_Glia
TGFBR1/Glutamatergic neurons	TGFB3/SLC41A1_Glia
ACVR2B/ Nitrergic neurons	TGFB3/SLC41A1_Glia
ACVR2B/ Nitrergic neurons	IGF1/SLC41A1_Glia
GNG8/ Nitrergic neurons	C5/SLC41A1_Glia
ANPEP/SPC24_Glia	ALB/SLC41A1_Glia
ANPEP/SPC24_Glia	VEGFA/ Nitrergic neurons
CALCRL/SLC41A1_Glia	VEGFA/ Nitrergic neurons

Supplementary Table 2

Coverage of functional linkages involving top five WikiPathways in the investigated cell types

Comparisons	Key DEG involved in the top five WikiPathways in the investigated cell types	Proportion of functional linkages involving the key DEG to those involving all DEG (%)
**p-pC-MG up vs p-dC-MG in porcine without VNS**	*MAPK3*, *PRKX*, *LDLR*	90.11
**p-pC-MG down vs p-dC-MG in porcine without VNS**	*GNG8*, *CXCL12*, *GUCY1A1*, *TGFBR3*	98.84
**p-pC-MG up vs p-dC-MG in porcine with VNS**	*CLTB*, *DAB2*, *MAPK3*, *NFKBIB*, *LYN*, *BCAR1*, *GRK5*, *PIK3CB*, *PRKX*, *GRK6*, *PTK2B*, *STAT1*, *EGR1*, *OSMR*, *LDLR*, *VEGFA*, *IL6ST*, *JAK1*, *ENG*, *TGIF1*	97.38
**p-pC-MG down vs p-dC-MG in porcine with VNS**	*TJP1*, *CXCL12*, *PIK3R1*, *TGFBR3*, *BMP4*	98.65

Note: p-pC-MG and p-dC-MG, myenteric ganglia in porcine proximal and distal colon, respectively; VNS, vagal nerve stimulation; up, upregulated expression; down, downregulated expression; DEG, differentially expressed gene.

Supplementary Table 3

Categories for the WikiPathways of interest

Synaptic plasticity	Neurotransmitter binding	Interactions between immune cells and neurons	Neuroprotection	Nitric Oxide	Neuroinflammation	Anti-inflammation	Cytokine production	Neurogenesis
Synaptic Vesicle Pathway	Synaptic Vesicle Pathway	Oncostatin M Signaling Pathway	NO/cGMP/PKG mediated Neuroprotection	Effects of Nitric Oxide		TGF-beta Receptor Signaling	Chemokine signaling pathway	Dopaminergic Neurogenesis
Glutamate binding, activation of AMPA receptors and synaptic plasticity	Glutamate binding, activation of AMPA receptors and synaptic plasticity	Cytokines and Inflammatory Response		Metabolism of nitric oxide: NOS3 activation and regulation				
Neurotransmitter receptors and postsynaptic signal transmission	Neurotransmitter receptors and postsynaptic signal transmission	COVID-19 Adverse Outcome Pathway		PI3K-Akt Signaling Pathway	PI3K-Akt Signaling Pathway		PI3K-Akt Signaling Pathway	
Neurotransmitter release cycle	Neurotransmitter release cycle							
Phosphodiesterases in neuronal function	Phosphodiesterases in neuronal function			Phosphodiesterases in neuronal function				
GABA receptor Signaling	GABA receptor Signaling							
Acetylcholine synthesis	Neurotransmitter uptake and metabolism In glial cells	Neurotransmitter uptake and metabolism In glial cells						
Cannabinoid receptor signaling	Acetylcholine binding and downstream events	Cannabinoid receptor signaling						
Nicotine Activity on Dopaminergic Neurons	Neurotransmitter clearance	Nicotine Activity on Dopaminergic Neurons						

Supplementary Table 4

Well-known cytokines and the receptors used for the analyses in our study

**Pro-inflammatory signaling**	
**IL-1**	IL1A, IL1B, IL18, IL33, IL36A, IL36B
**IL-6**	IL11, IL6R, CNTFR, CTF1, LIF, SPP1, OSM, OSMR
**TNFα**	TNF, LTA, TNFSF13B, TNFSF13
**IL-17**	IL17A, IL17B, IL17C, IL17D, IL17F, IL25
**IFN**	IFNB1, IFNAR1, IFNK, IFNL1
**C-C Motif chemokine**	CCR2, CCL2
**C-X-C Motif chemokine**	CXCL12
**Anti-inflammatory signaling**	
**IL-12**	IL12RB1, IL12RB2, IL23R, IL23A, IL27
**IL-10**	IL10, IL19, IL20, IL24, IL22, IL26
**TGFβ**	TGFB1, TGFBR1, TGFBR2, TGFBR3
**C-X-C Motif chemokine**	CXCL11

Supplementary Table 5

Summary of public GitHub repositories where R scripts were used for data analyses in our study

Name of the Repository	R packages	URL
BenaroyaResearch/DGETools<br>eonurk/RNA-seq-differential-analyses-guideline	edgeR<br>Limma-Voom	https://github.com/BenaroyaResearch/DGETools<br>https://github.com/eonurk/RNA-seq-differential-analyses-guideline
satijalab/seurat	Seurat	https://github.com/satijalab/seurat
rikenbit/scTensor	LRBase.Ssc.eg.db<br>DropletUtils<br>scTGIF<br>SingleCellExperiment<br>scTensor	https://github.com/rikenbit/scTensor
meichendong/SCDC	SCDC	https://github.com/meichendong/SCDC
danny-wilson/harmonicmeanp	harmonicmeanp	https://github.com/danny-wilson/harmonicmeanp
ssayols/rrvgo	rrvgo	https://github.com/ssayols/rrvgo/
