# Supplementary material for: Comparative transcriptomics reveals highly conserved regional programs between porcine and human colonic enteric nervous system
Source: Commun Biol. 2023 Jan 24;6:98. doi: 10.1038/s42003-023-04478-x (PMC9872754; doi:10.1038/s42003-023-04478-x)
Supplement: Supplementary file 3 — Description of Additional Supplementary Files [file 42003_2023_4478_MOESM3_ESM.pdf]

Description of Additional Supplementary Files

File name: Supplementary Data 1
Description: The source data behind the Figure 2a in the paper.

File name: Supplementary Data 2
Description: The source data behind the Figure 7 in the paper.

File name: Supplementary Data 3
Description: The source data behind the Figure 8 in the paper.

File name: Supplementary Data 4
Description: Source data for Figure 4c.

File name: Supplementary Data 5
Description: Source data for Figure 4d.
