# Supplementary material for: Comparative transcriptomics reveals highly conserved regional programs between porcine and human colonic enteric nervous system
Source: Commun Biol. 2023 Jan 24;6:98. doi: 10.1038/s42003-023-04478-x (PMC9872754; doi:10.1038/s42003-023-04478-x)
Supplement: Supplementary file 9 — Reporting Summary [file 42003_2023_4478_MOESM9_ESM.pdf]

nature portfolio

Corresponding author(s): Pu-Qing Yuan

Last updated by author(s): 12/22/2022

Reporting Summary

Nature Portfolio wishes to improve the reproducibility of the work that we publish. This form provides structure for consistency and transparency in reporting. For further information on Nature Portfolio policies, see our Editorial Policies and the Editorial Policy Checklist.

Statistics

For all statistical analyses, confirm that the following items are present in the figure legend, table legend, main text, or Methods section.

n/a	Confirmed	
☐	☒	The exact sample size (*n*) for each experimental group/condition, given as a discrete number and unit of measurement
☐	☒	A statement on whether measurements were taken from distinct samples or whether the same sample was measured repeatedly
☐	☒	The statistical test(s) used AND whether they are one- or two-sided<br>*Only common tests should be described solely by name; describe more complex techniques in the Methods section.*
☐	☒	A description of all covariates tested
☐	☒	A description of any assumptions or corrections, such as tests of normality and adjustment for multiple comparisons
☐	☒	A full description of the statistical parameters including central tendency (e.g. means) or other basic estimates (e.g. regression coefficient) AND variation (e.g. standard deviation) or associated estimates of uncertainty (e.g. confidence intervals)
☐	☒	For null hypothesis testing, the test statistic (e.g. *F*, *t*, *r*) with confidence intervals, effect sizes, degrees of freedom and *P* value noted<br>*Give P values as exact values whenever suitable.*
☒	☐	For Bayesian analysis, information on the choice of priors and Markov chain Monte Carlo settings
☐	☒	For hierarchical and complex designs, identification of the appropriate level for tests and full reporting of outcomes
☒	☐	Estimates of effect sizes (e.g. Cohen's *d*, Pearson's *r*), indicating how they were calculated

Our web collection on statistics for biologists contains articles on many of the points above.

Software and code

Policy information about availability of computer code

Data collection	The Sus_scrofa and Homo_sapiens genome index were created using Sus_scrofa genome file (Sscrofa11.1.fa) and annotation file (Sus_scrofa.Sscrofa11.1.95.gtf), and Homo_sapiens genome file (Homo_sapiens.GRCh38.dna.primary_assembly.fa) and annotation file (Homo_sapiens.GRCh38.97.gtf), respectively. These files were downloaded at https://uswest.ensembl.org /Sus_scrofa/Info/Index and https://uswest.ensembl.org/Homo_sapiens /Info/Index. The genome index was used by STAR v2.7.1a and Cell Ranger 3.1.0 for Sus_scrofa and by STAR v2.7.1a for Homo_sapiens. Gene homology search between human and porcine was performed, using ensemble multiple species comparison tool (http://www.ensembl.org/biomart/). The version of ENSEMBL BioMart is release 104.
Data analysis	Bulk RNA-seq data were processed and analyzed using STAR aligner v2.7.1a, R v3.6.2 packages edgeR v3.14 and Limma v3.14. Single-cell RNA-seq data were processed and analyzed using Cell Ranger 3.1.0 and R v3.6.2 package Seurat V3. Cell-cell interactions were inferred from the single-cell transcriptomic data using R v3.6.2 package scTensor v2.4.1. Images from single-molecule fluorescence in situ hybridization (smFISH) were visualized using Imaris 9.7.2 for Neuroscientists. Deconvolution of bulk RNA-seq data using single-cell RNA-seq datasets from naïve porcine colon was done using R v3.6.2 package SCDC. Calculation of standard RPKM (reads per kilobase of exon model per million mapped reads) expression values for the orthologous gene set was done using Python v3.8 package bioinfokit v0.9.1. R v3.6.2 packages ecdf and cor and Python v3.8 packages scipy.stats.ks_2samp and bioinfokit v0.9.5 were used for empirical cumulative density function (ECDF), cross-species Spearman's Correlation, Kolmogorov-Smirnov test and Pearson's Chi-squared test, respectively. Differentially expressed genes between porcine and human ENS were uncovered using R v3.6.2 package SCBN v1.12.0. Analysis of pathway enrichment using bulk and single-cell RNA sequencing data was done using g:Profiler (https://biit.cs.ut.ee/gprofiler/) or ClueGO v.2.5.6 or EnrichmentMap v.3.2.1 in Cytoscape v.3.8.2. R v3.6.2 package harmonicmeanp v3.0 was used to compute the FDR p-value. The bubble plots, heatmap and similarity matrix were generated using R v3.6.2 packages ggplot2, heatmap.2 and rrvgo v1.6.0, respectively.

For manuscripts utilizing custom algorithms or software that are central to the research but not yet described in published literature, software must be made available to editors and reviewers. We strongly encourage code deposition in a community repository (e.g. GitHub). See the Nature Portfolio guidelines for submitting code & software for further information.

Data

Policy information about availability of data

All manuscripts must include a data availability statement. This statement should provide the following information, where applicable:

- Accession codes, unique identifiers, or web links for publicly available datasets
- A description of any restrictions on data availability
- For clinical datasets or third party data, please ensure that the statement adheres to our policy

Raw and processed RNA sequencing data and sample metadata from this study have been deposited into the National Center for Biotechnology Information Gene Expression Omnibus under accession number: GSE197106, and publicly available on Feb 26, 2023.

Human research participants

Policy information about studies involving human research participants and Sex and Gender in Research.

Reporting on sex and gender	The full thickness of colonic specimens were dissected from healthy margin of the post-operative ascending, transverse and descending colon (h-aC, h-tC, h-dC, 4 of each) from 12 patients (5 males and 7 females). We did not perform sex-based analysis because the group size is small (two males and two females for h-aC and h-tC, one male and three females for h-dC), probably resulting in not solid conclusion. Therefore, in our study, we pooled the datasets from males and females.
Population characteristics	The full thickness of colonic specimens were dissected from healthy margin of the post-operative h-aC, h-tC and h-dC (4 of each) from 12 patients (5 males and 7 females, median age 47 years old, range 35-66 years) with colonic adenocarcinoma. None of the patients had active colonic infections when the tissues were collected.
Recruitment	All specimens were provided by the UCLA Translational Pathology Core Laboratory without identifiable private information after dissection and examination to be normal under macro- and microscopic inspection.
Ethics oversight	The use of human colon tissues was approved by UCLA Institutional Review Board for Biosafety and Ethics (IRB #17-001686). Informed consent has been obtained in all cases.

Note that full information on the approval of the study protocol must also be provided in the manuscript.

Field-specific reporting

Please select the one below that is the best fit for your research. If you are not sure, read the appropriate sections before making your selection.

☒ Life sciences ☐ Behavioural & social sciences ☐ Ecological, evolutionary & environmental sciences

For a reference copy of the document with all sections, see nature.com/documents/nr-reporting-summary-flat.pdf

Life sciences study design

All studies must disclose on these points even when the disclosure is negative.

Sample size	Sample size was based on available samples rather than on a pre-defined samples size calculation.
Data exclusions	For single-cell RNA-seq data analysis, we have used the following pre-established cell exclusion criteria: low quality cells were filtered out computationally (see Methods). The DEG were matched to the high-quality orthologous gene list, but we did not find any porcine orthologous DEG when comparing human ascending or descending colon with transverse colon. Therefore, only porcine proximal and distal colon were considered in the subsequent analyses.
Replication	At least three biological replicates for each experiments. We required replication at $p < 0.05$.
Randomization	The pigs were selected for vagal nerve stimulation group and control group entirely by chance with no regard to the will of researchers or animal condition and preference.
Blinding	Assignment of cells to different clusters for single-cell RNA-seq experiments was done using unsupervised clustering, which is not influenced by investigators.

Reporting for specific materials, systems and methods

We require information from authors about some types of materials, experimental systems and methods used in many studies. Here, indicate whether each material, system or method listed is relevant to your study. If you are not sure if a list item applies to your research, read the appropriate section before selecting a response.

Materials & experimental systems

n/a	Involved in the study
☒	☐ Antibodies
☒	☐ Eukaryotic cell lines
☒	☐ Palaeontology and archaeology
☐	☒ Animals and other organisms
☒	☐ Clinical data
☒	☐ Dual use research of concern

Methods

n/a	Involved in the study
☒	☐ ChIP-seq
☒	☐ Flow cytometry
☒	☐ MRI-based neuroimaging

Animals and other research organisms

Policy information about studies involving animals; ARRIVE guidelines recommended for reporting animal research, and Sex and Gender in Research

Laboratory animals	Six castrated male and three intact female Yucatan minipigs (~7 months old, 25-36 kg, S&S Farms, Ramona, CA) were fasted for 12 h and anaesthetized by intramuscular application of midazolam (1 mg/kg, cat # 067595, Covetrus, Dublin, OH), ketamine (15 mg/kg, cat # 068317, Covetrus, Dublin, OH) and meloxicam (0.3 mg/kg, #049755, Covetrus, Dublin, OH). Three out of the six male animals underwent electrical stimulation of the celiac branch of the abdominal vagus nerve (2 Hz, 0.3-4 ms, 5 mA, 10 min) using pulse train. A detailed experimental protocol for VNS is available at https://www.protocols.io/view/tache-mulugeta-ot2od024899-colon-tissue-electrical-3rmgm.
Wild animals	The study did not use wild animals.
Reporting on sex	Because the vendor does not provide the normal animals, we used six castrated male and three intact female Yucatan minipigs for analyses. We found some sex differences, but we cannot figure out if the differences originate truly from the sex due to the use of the castrated male pigs. Therefore, in our study, we pooled the datasets from males and females.
Field-collected samples	The study did not involve field-collected samples.
Ethics oversight	All animal care and procedures were performed following National Institutes of Health guidelines for the humane use of animals, with approval and in accordance with the guidelines of the University of California at Los Angeles (UCLA) Institutional Animal Care and Use Committee, Chancellor's Animal Research Committee (ARC) (protocol 2018-074-01). All efforts were made to avoid suffering.

Note that full information on the approval of the study protocol must also be provided in the manuscript.
